# Predicting distant metastasis of bladder cancer using multiple machine learning models: a study based on the SEER database with external validation

**DOI:** 10.3389/fonc.2024.1477166

**Published:** 2024-12-13

**Authors:** Xin Chang Zou, Xue Peng Rao, Jian Biao Huang, Jie Zhou, Hai Chao Chao, Tao Zeng

**Affiliations:** ^1^ The Second Affiliated Hospital, Jiangxi Medical College, Nanchang University, Nanchang, China; ^2^ Department of Urology, Second Affiliated Hospital of Nanchang University, Nanchang, China

**Keywords:** machine learning, bladder cancer, SEER database, distant metastasis, predictive value

## Abstract

**Background and purpose:**

Distant metastasis in bladder cancer is linked to poor prognosis and significant mortality. Machine learning (ML), a key area of artificial intelligence, has shown promise in the diagnosis, staging, and treatment of bladder cancer. This study aimed to employ various ML techniques to predict distant metastasis in patients with bladder cancer.

**Patients and methods:**

Patients diagnosed with bladder cancer in the Surveillance, Epidemiology, and End Results (SEER) database from 2000 to 2021 were included in this study. After a rigorous screening process, a total of 4,108 patients were selected for further analysis, divided in a 7:3 ratio into a training cohort and an internal validation cohort. In addition, 118 patients treated at the Second Affiliated Hospital of Nanchang University were included as an external validation cohort. Features were filtered using the least absolute shrinkage and selection operator (LASSO) regression algorithm. Based on the significant features identified, three ML algorithms were utilized to develop prediction models: logistic regression, support vector machine (SVM), and linear discriminant analysis (LDA). The predictive performance of the three models was evaluated by obtaining the area under the receiver operating characteristic (ROC) curve (AUC), the precision, the accuracy, and the F1 score.

**Results:**

According to the statistical results, the final probability of distant metastasis in the population was 12.0% (*n* = 495). LASSO regression analysis revealed that age, chemotherapy, tumor size, the examination of non-regional lymph nodes, and regional lymph node evaluation were significantly associated with distant metastasis of bladder cancer. In the internal validation cohort, the prediction accuracy rates for logistic regression, SVM, and LDA were 0.874, 0.877, and 0.845, respectively. The precision rates were 0.805, 0.769, and 0.827, respectively, and the F1 scores were 0.821, 0.819, and 0.835, respectively. The ROC curve demonstrated that the AUC for all models was greater than 0.7. In the external validation cohort, the prediction accuracy rates for logistic regression, SVM, and LDA were 0.856, 0.848, and 0.797, respectively, with the ROC curve indicating that the AUC also exceeded 0.7. The precision rates were 0.877, 0.718, and 0.736, respectively, and the F1 scores were 0.797, 0.778, and 0.762, respectively. Among the algorithms used, logistic regression demonstrated better predictive efficiency than the other two methods. The top three variables with the highest importance scores in the logistic regression were non-regional lymph nodes, age, and chemotherapy.

**Conclusion:**

The prediction model developed using three ML algorithms demonstrated strong accuracy and discriminative capability in predicting distant metastasis in patients with bladder cancer. This might help clinicians in understanding patient prognosis and in formulating personalized treatment strategies, ultimately improving the overall prognosis of patients with bladder cancer.

## Introduction

Bladder cancer (Bca) is one of the most common malignant tumors of the urinary tract. As the ninth most prevalent malignant tumor globally, its incidence and prevalence are increasing year by year. Approximately 550,000 patients are newly diagnosed with Bca each year (1, 2). Men tend to have a higher incidence, and smoking is believed to be a contributing factor (3). Based on the ability of the tumor to invade, Bca can be classified into non-muscle-invasive bladder cancer (NMIBC), muscle-invasive bladder cancer (MIBC), and metastatic forms of the disease (4). Approximately 70% of newly diagnosed cases are NMIBC, while approximately 30% are diagnosed as MIBC, frequently with metastatic characteristics. Half of the patients with muscle-invasive disease will die from metastases within 2 years. Compared with the 5-year survival rate of 77% for all stages of Bca, the 5-year survival rate for metastatic Bca is only 5% (5–7). Therefore, it is critical to develop models for the prediction of distant metastasis of Bca.

As an essential branch of artificial intelligence (AI), machine learning (ML) develops predictive models by automatically learning from large datasets to improve prediction algorithms. This advancement assists clinicians in identifying high-risk patients and in evaluating the prognosis of various diseases (8, 9). Diagnostic and prognostic models built using ML algorithms based on pathomic data have demonstrated remarkable efficiency in distinguishing patients with Bca from those with glandular cystitis, as well as in predicting the survival outcomes of BCa (10). Network analysis methods rooted in ML frameworks can effectively identify biomarkers linked to immunotherapy responses, resulting in robust predictions for precision oncology (11). Furthermore, a ML model utilizing full-sequence MRI can accurately forecast the depth of invasion of Bca prior to surgery, helping clinicians in recognizing pathological features associated with tumor invasion ahead of invasive procedures (12). In addition, ML has been widely applied in prostate cancer and kidney cancer research, and it also holds significant potential in various benign conditions, such as urinary tract stones (12, 13).

In this study, data on patients with Bca and their clinical and pathological characteristics were retrieved from the Surveillance, Epidemiology, and End Results (SEER) database from 2000 to 2021. Our aim was to utilize ML algorithms to develop a reliable model for the prediction of distant metastasis of Bca, thereby providing clinical support for treatment decisions and individualized prognosis assessment.

## Patients and methods

### Patients

**Figure 1:**
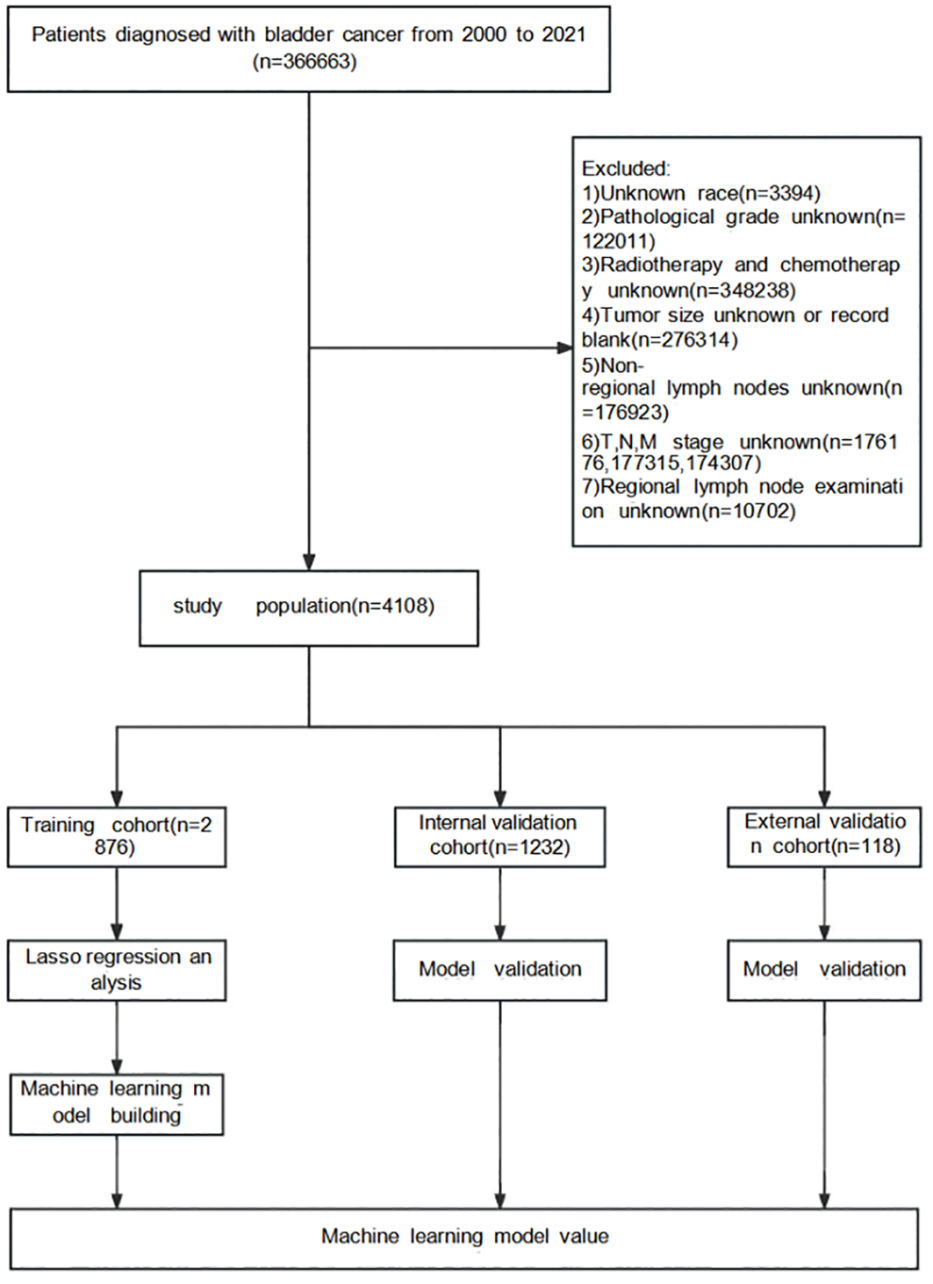
Surveillance, Epidemiology, and End Results (SEER) database screening and research flowchart.

Detailed data of patients with Bca from 17 registration centers between 2000 and 2021 were collected from SEER*Stat (version 8.4.3), including the demographic, clinical, and pathological characteristics. The inclusion criterion comprised patients diagnosed with Bca during this time frame. The exclusion criteria were cases with unknown race, unknown pathological grade, unknown radiotherapy status, unknown tumor size or blank records, unknown non-regional lymph nodes, and unknown T, N, or M stage, as well as unknown regional lymph node examination results. **Figure 1** illustrates the complete screening process. As SEER is a public database and all records are de-identified, no additional ethical approval was required.

The external validation cohort comprised 118 eligible patients diagnosed with Bca between 2016 and 2020. Upon review and analysis, a total of 18 patients were found to have ultimately developed distant metastasis. The final follow-up was scheduled for December 2023. This study was approved by the Ethics Committee of the Second Affiliated Hospital of Nanchang University, with a waiver of informed consent.

### Screening for clinical and pathological features

The variables selected included demographic characteristics (i.e., age, sex, and race), tumor characteristics (i.e., tumor size, grade, histology, T stage, and N stage), treatment information (e.g., chemotherapy and radiation therapy), and other variables (e.g., regional lymph nodes examined and non-regional lymph nodes). The primary endpoint of this study was the development of distant metastases in Bca. For better analysis, some of the variables were processed in the SEER database. A number of continuous variables, i.e., age, tumor size, regional lymph nodes examined, and non-regional lymph nodes, were transformed into categorical variables. All clinical and pathological features were used in the training set to screen the features most related to the distant metastasis of Bca using least absolute shrinkage and selection operator (LASSO) regression.

Variables were selected not only for their individual significance but also for their potential interactions and contributions to the overall predictive model. The LASSO regression technique applies a penalty proportional to the absolute value of the coefficient sizes, effectively shrinking some coefficients to zero. This process facilitates variable selection while preventing overfitting, which is particularly important when dealing with high-dimensional datasets. The tuning parameter (*λ*) regulates the strength of the penalty: a larger *λ* leads to more coefficients being set to zero, thereby highlighting the most important predictors.

The clinical rationale for selecting these variables is grounded in their established correlations with outcomes in previous studies and clinical guidelines. For instance, age and gender have been shown to significantly influence the prognosis of cancer and the response to treatment. Tumor characteristics such as size, grade, and stage (T and N) are critical in the assessment of the aggressiveness of a cancer and the likelihood of metastasis. In addition, treatment variables, particularly the effects of chemotherapy and radiation therapy on cancer progression, have been well documented and are essential for our analysis. Lastly, examination of regional and non-regional lymph nodes is crucial for the assessment of the metastatic spread, thus helping in the development of predictive models for distant metastasis.

### Construction of the machine learning model

As a common linear classifier, logistic regression establishes a regression formula for the classification boundary based on existing data, enabling the accurate identification of binary classification problems. It primarily implements the algorithm through the logistic function, specifically the sigmoid function. Support vector machine (SVM), as a binary classification model, is fundamentally defined as a linear classifier that maximizes the margin in the feature space. By maximizing this margin, SVM can ultimately be transformed into a solution for a convex quadratic programming problem. Linear discriminant analysis (LDA), a classic method in statistics and ML, aims to maximize the separation between samples of different categories while minimizing the scatter within the same category through a projection method, thus achieving linear classification of data.

The clinical and pathological characteristics identified above were utilized in constructing the ML models. Three types of ML algorithms were trained using a binary classification approach. The training set (70%) was used to train the model, while the validation set (30%) was employed to assess the predictive performance of the model, followed by external validation. A weighted average of the precision, accuracy, and F1 score was employed to minimize the impact of sample imbalance on the evaluation results. The predictive values of logistic regression, SVM, and LDA were compared by calculating the prediction accuracy, precision, F1 score, and the area under the receiver operating characteristic (ROC) curve (AUC).

### Statistical analysis

Data analysis was conducted using SPSS 27.0, R language software (version 4.3.1; http://www.r-project.org/), and Python language software (https://www.python.org/downloads/release/python-380/). For the analysis and processing, all categorical variables were expressed as numbers with percentages, and LASSO regression was employed to identify key predictors. LASSO regression was carried out using the glmnet package in R or the scikit-learn package in Python, and the ROC curve was plotted using the matplotlib package in Python.

## Results

### Patient characteristics and metastasis

The characteristics of the training cohort, the internal validation cohort, and the external validation cohort of patients are presented in **Table 1**. The probabilities of distant metastasis for Bca in the three cohorts were 11.9% (*n* = 343), 12.3% (*n* = 152), and 19.4% (*n* = 14), respectively.

**Table 1:** Clinical and pathological characteristics of the patients across the three cohorts.

Variables	Training (*n* = 2,876)	Internal validation (*n* = 1,232)	External validation (n = 118)
Distant metastasis, *n* (%)			
No	2,533 (88.07)	1,080 (87.66)	100 (84.75)
Yes	343 (11.93)	152 (12.34)	18 (15.25)
Age, *n* (%)			
<60 years	298 (10.36)	139 (11.28)	16 (13.56)
60–80 years	1,352 (47.01)	611 (49.59)	55 (46.61)
>80 years	1,226 (42.63)	482 (39.12)	47 (39.83)
Gender, *n* (%)			
Men	2,050 (71.28)	832 (67.53)	81 (68.64)
Women	826 (28.72)	400 (32.47)	37 (31.36)
Race, *n* (%)			
White	2,512 (87.34)	1,061 (86.12)	0
Black	158 (5.49)	162 (13.15)	0
Other	206 (7.16)	9 (0.73)	118 (100)
Histology, *n* (%)			
TCC	2,483 (86.34)	1,049 (85.15)	98 (83.05)
NTCC	393 (13.66)	183 (14.85)	20 (16.95)
Grade, *n* (%)			
Grade I	34 (1.18)	17 (1.38)	18 (15.25)
Grade II	150 (5.22)	73 (5.93)	6 (5.09)
Grade III	833 (28.96)	464 (37.66)	36 (30.51)
Grade IV	1,859 (64.64)	678 (55.03)	58 (49.15)
Radiotherapy, *n* (%)			
No	160 (5.56)	53 (4.30)	79 (66.95)
Yes	2,716 (94.44)	1,179 (95.70)	39 (33.05)
Chemotherapy, *n* (%)			
No	1,074 (37.34)	495 (40.18)	47 (39.83)
Yes	1,802 (62.66)	737 (59.82)	71 (60.17)
Tumor size, *n* (%)			
≥3 cm	2,304 (80.11)	998 (81.01)	95 (80.51)
<3 cm	572 (19.89)	234 (18.99)	23 (19.49)
Non-regional lymph nodes, *n* (%)			
No	2,502 (87.00)	1,047 (84.98)	102 (86.44)
Yes	374 (13.00)	185 (15.02)	16 (13.56)
T stage, *n* (%)			
T1	314 (10.92)	150 (12.18)	12 (10.17)
T2	1,801 (62.62)	751 (60.96)	84 (71.19)
T3	406 (14.12)	146 (11.85)	12 (10.17)
T4	355 (12.34)	185 (15.02)	10 (8.47)
N stage, *n* (%)			
N0	2,522 (87.69)	1,053 (85.47)	61 (51.69)
N1	189 (6.57)	73 (5.93)	20 (16.95)
N2	157 (5.46)	103 (8.36)	19 (16.10)
N3	8 (0.28)	3 (0.24)	18 (15.26)
Regional nodes examined, *n* (%)			
<10	2,663 (92.60)	1,168 (94.81)	111 (94.07)
10–20	137 (4.76)	37 (3.00)	3 (2.54)
>20	76 (2.64)	27 (2.19)	4 (3.39)

“Other” included American Indian/Alaska Native/Asian/Pacific Islander.

*TCC*, transitional cell carcinoma; *NTCC*, non-transitional cell carcinoma

### Identification of significant features

**Figure 2:**
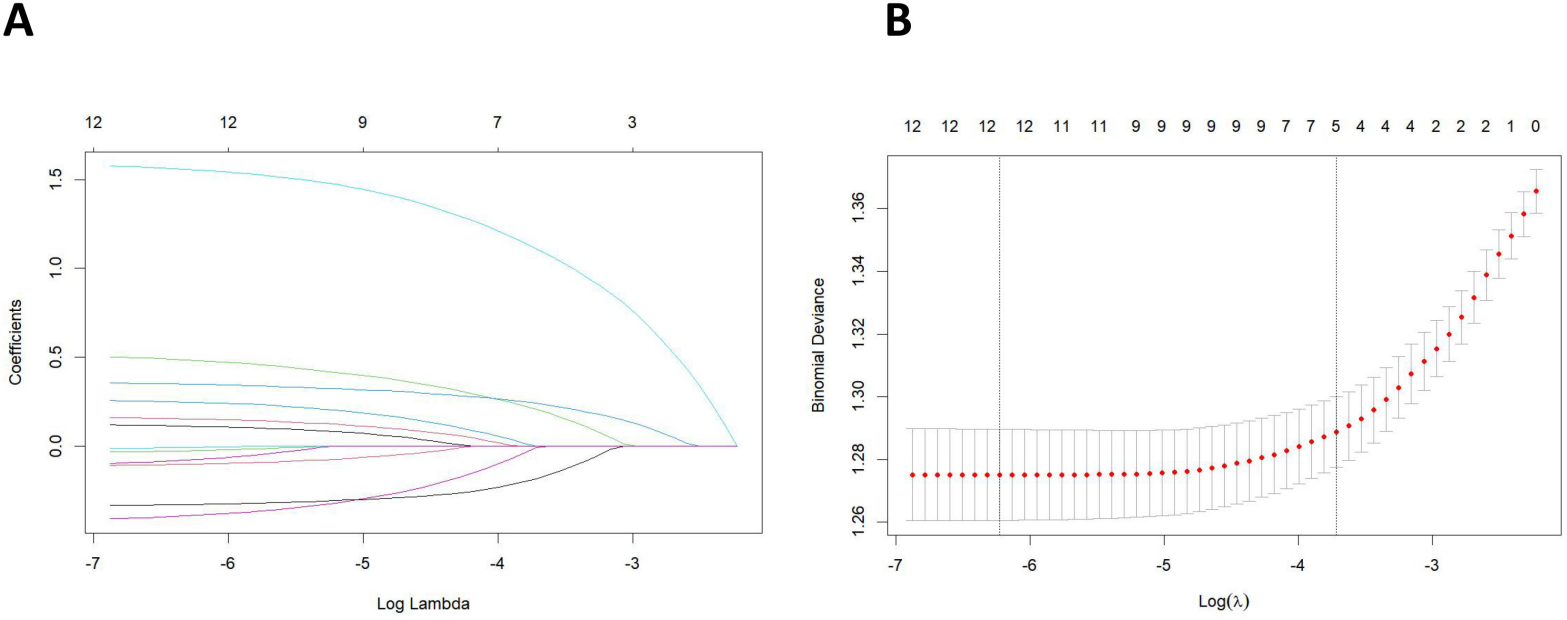
**(A)** Least absolute shrinkage and selection operator (LASSO) coefficient distribution of the clinical and pathological characteristics. **(B)** LASSO regression analysis was performed using the minimum criterion and a 10-fold cross-validation method. By introducing a penalty adjustment parameter (*λ*), the coefficients of the features are compressed towards zero, enabling automatic feature selection.

All clinical and pathological characteristics were analyzed using LASSO regression. The results indicated that age, chemotherapy, tumor size, non-regional lymph nodes, and regional lymph node examination were the significant variables associated with the distant metastasis of Bca, as detailed in **Figure 2**.

### Construction and evaluation of machine models

**Figure 3:**
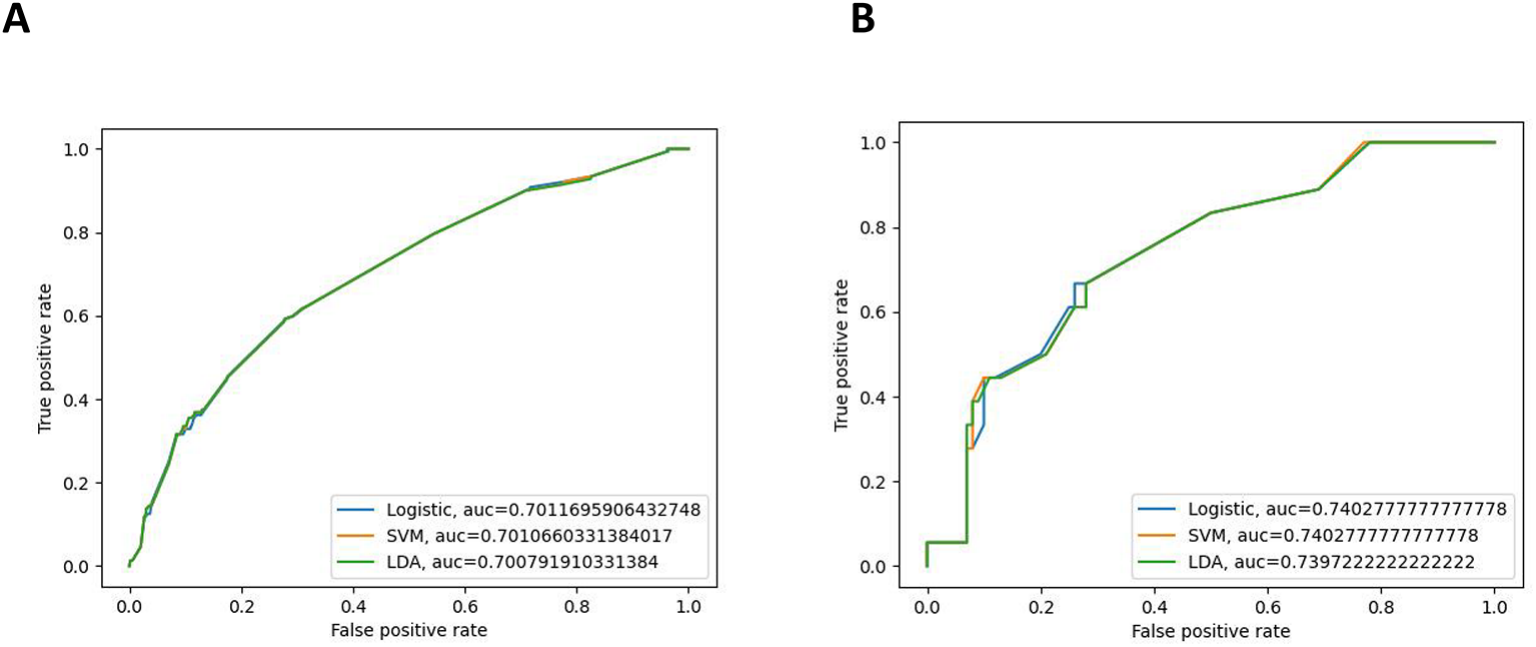
Receiver operating characteristic (ROC) curves of the internal validation cohort **(A)** and the external validation cohort **(B)**.

**Figure 4:**
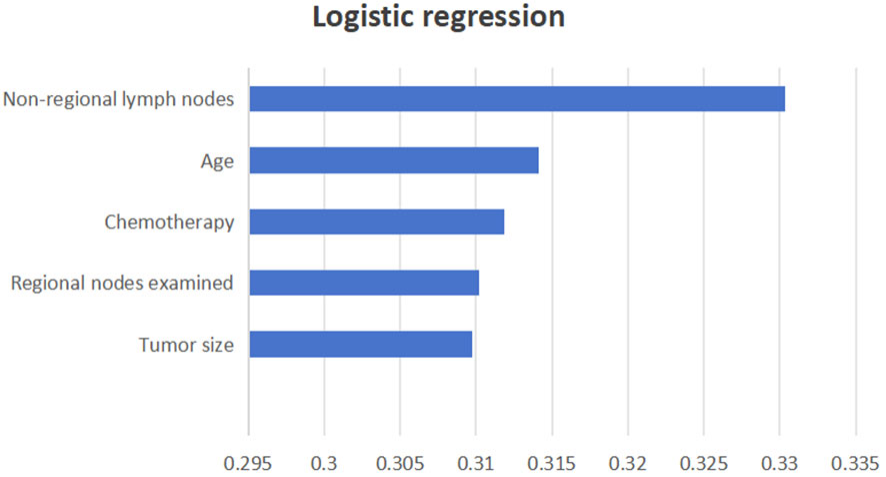
Logistic regression importance scores.

Three ML models were developed using the significant variables identified earlier. A weighted average of the precision, accuracy, and the F1 score was utilized to minimize the effects of sample imbalance on the evaluation results. In the internal validation cohort, the prediction accuracy rates for logistic regression, SVM, and LDA were 0.874, 0.877, and 0.845, respectively. The accuracy rates were 0.805, 0.769, and 0.827, respectively, and the F1 scores were 0.821, 0.819, and 0.835, respectively. The AUC (95% CI) values were 0.701 (0.634–0.767), 0.701 (0.641–0.766), and 0.701 (0.635–0.780), respectively. In the external validation cohort, the prediction accuracy rates for logistic regression, SVM, and LDA were 0.856, 0.848, and 0.797, respectively. The precision rates were 0.877, 0.718, and 0.736, respectively, and the F1 scores were 0.797, 0.778, and 0.762, respectively. The AUC (95% CI) values were 0.740 (0.682–0.795), 0.740 (0.674–0.792), and 0.740 (0.685–0.796), respectively. Details can be found in **Figure 3**. These results indicate that all three models can effectively predict distant metastasis in Bca. However, after comprehensive comparison, logistic regression was found to have the best predictive value and identification ability. The top three variables with logistic regression importance scores were non-regional lymph nodes, age, and chemotherapy (**Figure 4**).

## Discussion

Distant metastasis of Bca significantly impacts the survival rates and treatment prospects of patients. The common sites of metastasis include the liver, kidneys, and bones. Patients with Bca face an increased risk of death due to distant metastasis (14, 15). Those with localized Bca often experience an excellent quality of life and progression-free survival with aggressive treatment. Despite advancements in various targeted therapies and immunotherapies, distant metastasis remains highly lethal when it occurs (16–18). The metastatic spread in patients with Bca is believed to be random and unpredictable. Although early systemic treatment for Bca has made progress and is somewhat effective in preventing tumor progression, some individuals still inevitably develop recurrent or metastatic disease (19, 20). Therefore, it is crucial to develop new models for predicting the metastasis of Bca.

ML, a subfield of AI, emerged from the intersection of statistics and computer science. Among the algorithms that demonstrate superior performance, LDA and SVM are the most commonly recognized (21, 22). The continuous advancement of computing systems and recognition software has contributed to the popularity of ML-based systems. These systems are capable of performing complex tasks in bioinformatics and medical imaging, assisting healthcare professionals in improving the diagnostic accuracy, predicting disease progression, and even exploring new therapeutic avenues (23–25). A retrospective study by Denget al. showed that a ML model combining radiomic features and clinical variables could accurately predict the pathological grade of Bca (26). In addition, the quantitative assessment of tumor-infiltrating lymphocytes using ML mitigates the inter- and intra-observer variability, effectively predicting survival in MIBC and identifying patients who may benefit from immunotherapy or treatment adjustments (27). Liu et al. predicted lung metastasis in Bca by developing a nomogram model; however, this model did not include all distant metastases and highlighted the need for improved algorithmic approaches (28). In the study by Chen et al., multiple clinical variables were utilized to develop a nomogram for predicting distant metastasis in patients with urothelial Bca (29). In this research, a more advanced ML algorithm was employed, incorporating AI methods to successfully predict distant metastasis in Bca. ML utilizes advanced algorithms to analyze various clinical variables and integrate multiple data sources, resulting in a more comprehensive assessment of the condition of a patient. This approach improves the diagnostic accuracy and reduces the likelihood of misdiagnosis (9).

Our research utilized three ML models: logistic regression, SVM, and LDA. After screening the clinical variables and pathological characteristics using LASSO regression, we incorporated the identified significant variables and features into the construction of our ML models. In the internal validation cohort, we evaluated the predictive capability of the three models by calculating the prediction accuracy, precision, F1 score, and the AUC. The results indicated that all three models effectively predicted distant metastasis in Bca. This was further confirmed with the external validation cohort. By utilizing the model described above, the stage and the pathological type of patients with Bca can be predicted in real time, allowing for targeted treatment and follow-up for those at high risk of distant metastasis. However, this study has several limitations. Firstly, as a retrospective analysis based on the SEER database, it may have lacked certain potentially important factors, such as vascular invasion and preoperative laboratory results. In addition, patients with unknown variable information were excluded, which means that the results were inevitably impacted by selection bias. Secondly, variations in the sequencing of radiotherapy and chemotherapy, the types of radiation used, and the chemotherapy agents administered may have affected the likelihood of Bca metastasizing to distant sites. Finally, while we have validated our model using an external cohort, it is essential to include additional clinical and molecular biological features in the future to enhance its predictive power in complex clinical settings. Furthermore, multicenter prospective studies are essential for the development of more robust algorithms, such as deep learning and ensemble models. These studies should also focus on comparing model performance across different groups (such as gender and age) in multicenter external validations to further verify our findings.

## Conclusion

In summary, we successfully developed and validated three ML models for the prediction of distant metastasis in Bca. In particular, the logistic regression algorithm achieved better prediction results. The application of these models can assist clinicians in making more accurate treatment decisions and in providing personalized follow-up management, ultimately improving patient prognosis.

## Data Availability

The raw data supporting the conclusions of this article will be made available by the authors, without undue reservation.
